# Supplementary material for: Heat adaptation of phage T7 under an extended genetic code
Source: Virus Evol. 2021 Dec 1;7(2):veab100. doi: 10.1093/ve/veab100 (PMC8923235; doi:10.1093/ve/veab100)
Supplement: veab100_Supp [file veab100_supp.zip › Sequencing_Analysis.zip!/Sequencing_Analysis/A3/evidence/SNP_3.html]

BRESEQ :: Evidence


| Predicted mutation | | | | | | |
| --- | --- | --- | --- | --- | --- | --- |
| evidence | seq id | position | mutation | annotation | gene | description |
| RA | NC\_001604.1 | 15,094 | G→T | intergenic (–/–) | – / – | –/– |

| Read alignment evidence... | | | | | | | | | | | |
| --- | --- | --- | --- | --- | --- | --- | --- | --- | --- | --- | --- |
|  | seq id | position | | ref | new | freq | score (cons/poly) | reads | annotation | genes | product |
| \* | NC\_001604.1 | 15,094 | 0 | G | T | 99.4% | 4047.1 / NA | 1779 | intergenic (–/–) | *–/–* | –/– |
| Reads supporting (aligned to +/- strand):  **ref** base G (0/10);  **new** base T (903/860);  **total** (905/874) | | | | | | | | | | | |
| Fisher's exact test for biased strand distribution *p*-value = 7.88e-04 | | | | | | | | | | | |
| Kolmogorov-Smirnov test that lower quality scores support variant *p*-value = 1.00e+00 | | | | | | | | | | | |

Only 100 of 1789 total aligned reads displayed.  

`AGAGCAGGGTGAAGAATACGTTGACGGAATGGAGTGGTGGAACTTCAACGAAGAGATGATGGACTATAACGTTCAGGACGTTGTGGTAACTAAAGCTCTCCTTGAGAAGCTACTCTCTGACAAACATTACTTCCCTCCTGAGATTGACTTTACGGACGTAGGATACACTACGTTCTGGTCAGAATCCCTTGAGGCCGTTGACATTGAACATCGTGCTGCATGGCTGCTCGCTAAACAAGAGCGCAACGGGTTCCCGTTTGACACAAAAGCAATCGAAGAGTTGTACGTAGAGTTAGCTGCTCGCCGCTCTGAGTTGCTCCGTAAATTGACCGAAACGTTCGGCTCGTGGTATCAGCCTAAAGGTGGCACTGAGATGTTCTGCCATCCGCGAACAGGTAAGCCACTACCTAAATACCCTCGCATTAAGACACCTAAAGTTGGTGGTATCTTTAAGAAGCCTAAGAACAAGGCACAGCGAGAAGGCCGTGAGCCTTGCGAACTTGATACCCGCGAGTACGTTGCTGGTGCTCCTTACACCCCAGTTGAACATGTTGTGTTTAACCCTTCGTCTCGTGACCACATTCAGAAGAAACTCCA  >  NC_001604.1/14796‑15392`  

                                                                                                                                                                                                                                                                                                          `|`                                                                                                                                                                                                                                                                                                            
      `gggTGAAGAATACGTTGACGGAATGGAGTGGTGGAACTTCAACGAAGAGATGATGGACTATAACGTTCAGGACGTTGTGGTAACTAAAGCTCTCCTTGAGAAGCTACTCTCTGACAAACATTACTTCCCTCCTGAGATTGACTTTACGGACGTAGGATACACTAAGTTCTGGTCAGAATCCCTTGAGGCCGTTGACATTGCACATCGTGCTGTATGGCTGCTCGCTAAACAAGAGCGCAACGGGTTCCCGTTTGACACAAAAGCAATCGAAGACGTGTACGTAGAGTTAGCTTCTtgccgc                                                                                                                                                                                                                                                                                                    >  1:243754/1‑301 (MQ=255)`  
       `ggTGAAGAATACGTTGACGGAATGGAGTGGTGGAACTTCAACGAAGAGATGATGGACTATAACGTTCAGGACGTTGTGGTAACTAAAGCTCTCCTTGAGAAGCTACTCTCTGACAAACATTACTTCCCTCCTGAGATTGACTTTACGGACGTAGGATACACTACGTTCTGGTCAGAATCCCTTGAGGCCGTTGACATTGAACATCGTGCTGCATGGCTGCTCGCTAAACAAGAGCGCAACGGGTTCCCGTTTGACACAAAAGCAATCGAAGAGTTGTACGTAGAGTTAGCTTCTCGCCGct                                                                                                                                                                                                                                                                                                   >  1:125537/1‑301 (MQ=255)`  
        `gTGAAGAATACGTTGACGGAATGGAGTGGTGGAACTTCAACGAAGAGATGATGGACTATAACGTTCAGGACGTTGTGGTAACTAAAGCTCTCCTTGAGAAGCTACTCTCTGACAAACATTACTTCCCTCCTGAGATTGACTTTACGGACGTAGGATACACTACGTTCTGGTCAGAATCCCTTGAGGCCGTTGACATTGAACATCGTGCTGCATGGCTGCTCGCTAAACAATAGCGCAACGGGTTCCCGTTTGACACAAAAGCAATCGAAGAGTGTTACGTAGAGTTAGCTTCTCGCCGctt                                                                                                                                                                                                                                                                                                  >  1:118193/1‑300 (MQ=255)`  
        `aatgtgtataagagacaggAATGGAGTGGTGGAACTTCAACGAAGAGAGGATGGACTATAACGTTCAGGACGTTGTGGTAACTAAAGCTCTCCTTGAGAAGCTACTCTCTGACAAACATTACTTCCCTCCTGAGATTGACTTTACGGACGTAGGATACACTACGTTCTGGTCAGAATCCCTTGAGGCCGTTGACATTGAACATCGTGCTGCATGGCTGCTCGCTAAACAAGAGCGCAACGGGTTCCCGTTTGACACAAAAGCAATCGAAGAGTTGTACGTAGAGTTAGCTTCCCGCCGctc                                                                                                                                                                                                                                                                                                  <  1:2205/284‑1 (MQ=255)`  
          `gaagaaTACGTTGACGGAATGGAGTGGTGGAACTTCAACGAAGAGATGATGGACTATAACGTTCAGGACGTTGTGGTAACTAAAGCTCTCCTTGATAAGCTACTCTCTGACAAACATTACTTCCCTCCTGAGATTGACTTTACGGACGTAGGATACACTACGTTCTGGTCAGAATCCCTTGAGGCCGTTGACATTGAACATCGTGCTGCATGGCTGCTCGCTAAACAAGAGCGCAACGGGTTCCCGTTTGACACAAAAGCAATCGAAGAGTTGTACGTAGCGTTAGCTTCTCGCCGCTCTg                                                                                                                                                                                                                                                                                                >  1:71703/1‑301 (MQ=255)`  
                     `tGACGGAATGGAGTGGTGGAACTTCAACGAAGAGATGATGGACTATAACGTTCAGGACGTTGTGGTAACTAAAGCTCTCCTTGAGAAGCTACTCTCTGACAAACATTACTTCCCTCCTGAGATTGACTTTACGGACGTAGGATACACTACGTTCTGGTCAGAATCCCTTGAGGCCGTTGACATTGAACATCTTGCTGCATGGCTGCTCGCTAAACAAGAGCGCAACGGGTTCCCGTTTGACACAAAAGCAATCGAAGAGTTGTACGTAGAGTTAGCTTCTCGCCGCCCTTAGTTGCTCCGt                                                                                                                                                                                                                                                                                     >  1:200006/1‑301 (MQ=255)`  
                       `aCGGAATGGAGTGGTGGAACTTCAACGAAGAGATGATGGACTATAACGTTCAGGACGTTGTGGTAACTAAAGCTGTCCTTGAGAAGCTACTCTCTGACAAACATTACTTCCCTCCTGAGATTGACTTTACGGACGTAGGATACACTACGTTCTGGTCAGAATCCCTTGAGGCCTTTGACATTGAACATCGTGCTGCATGGCTGATCGCTAAACAAGAGCGCAACGGGTTCCCGTTTGACACAAAAGCAATCGAAGAGTTGTACGTAGAGTTAGCTTCTCGCCGCTCTGAGTTGCTCCGTaa                                                                                                                                                                                                                                                                                   <  1:43407/301‑1 (MQ=255)`  
                         `ggAATGTAGTGGTGGAACTTCAACGAAGAGATGATGGACTATAACGTTCAGGACGTTGTGGTAACTAAAGCTCTCCTTGAGAAGCTACTCTCTGACAAACATTACTTCCCTCCTGAGATTGACTTTACGGACGTAGGATACACTACGTTCTGGTCAGAATCCCTTGAGGCCGTTGACATTGAACATCGTGCTGCATGGCTGCTCGCTAAACAAGAGCGCAACGGGTTCCCGTTTGACACAAAAGCAATCGAAGAGTTGTACGGAGAGTTAGCTTCTCGCCGCTCTGAGTTGCCCCGTAAAt                                                                                                                                                                                                                                                                                 >  1:226905/1‑301 (MQ=255)`  
                              `ggAGTGGTGGAACTTCAACTAAGAGATGATGGACTATAACGTTCAGGACGTTGTGGTAACTAAAGCTCTCCTTGAGAAGCTACTCTCTGACAAACATTACTTCCCTCCTGAGATTGACTTTACGGACGTAGGATACACTACGTTCTGGTCAGAATCCCTTGAGGCCGTTGACATTGAACATCGTGCTGCATGGCTGCTCGCTAAACAAGAGCGCAACGGGTTCCCGTTTGACACAAAAGCAATCGAAGAGTTGTACGTAGAGTTAGCTTCTCGCCGCTCTGAGTTGCTCCGTAAATTGAcc                                                                                                                                                                                                                                                                            <  1:26219/301‑1 (MQ=255)`  
                                              `aaCGAAGAGATGATGGACTAGAACGTTCAGGACGTTGTGGTAACTAAAGCTCTCCTTGAGAAGCTACTCTCTGACAAACATTACTTCCCTCCTGAGATTGACTTTACGGACGTATGATACACTACGTTCTGGTCAGAATCCCTTGAGGCCGTTGACATTGAACATCGTGCTGCATGGCTGCTCGCTAAACAAGAGCGCAACGGGTTCCCGTTTGACACAAAAGCAATCGAAGAGTTGTACGTAGAGTTAGCTTCACGCCGCTCTGAGTTGCTCCGTAAATTGACCGAAACGTTCGGCTCGt                                                                                                                                                                                                                                                            <  1:74309/301‑1 (MQ=255)`  
                                                `gagacagaTGATGGACTATAACGTTCAGGACGTTGTGGTAACTAAAGCTCTCCTTGAGAAGCTACTCTCTGACAAACATTACTTCCCTCCTGAGATTGACTTTACGGACGTAGGATACACTACGTTCTGGTCAGAATCCCTTGAGGCCGTTGACATTGAACATCGTGCTGCATGGCTGCTCTCTAAACAAGAGCGCAACTGGTTCCCGTTTGACACAAAAGCAATCGAAGAGTTGTACGTAGAGTTAGCTTCTCGCCGCTCTGAGTTGCTCCGTAAATTGACCGAAACGTTCGGCTCGTgg                                                                                                                                                                                                                                                          <  1:121924/296‑1 (MQ=255)`  
                                                        `tgatgGACTATAACGTTCAGGACGTTGTGGTAACTAAAGCTCTCCTTGAGAAGCTACTCTCTGACAAACATTACTTCCCTCCTGAGATTGACTTTACGGACGTAGGATACACTACGTTCTGGTCAGAATCCCTTGAGGCCGTTGACATTGAACATCGTGCTGCATGGCTGCTCGCTAAACAAGAGCGCAACGGGTTCCCGTTTGACACAAAAGCAATCGAAGAGTTGTACGTAGAGTTAGCTTCTCGCCGCCCTGAGTTGCTCCGTAAATTGACCGAAACGTTCGGCTCGTGGTATCAGcc                                                                                                                                                                                                                                                  >  1:272429/1‑301 (MQ=255)`  
                                                        `acagggACTATAACGTTCATGACGTTGTGGTAACTAAAGCTCTCCTTGAGAAGCTACTCTCTGACAAACATTACTTCCCTCCTGAGATTGACTTTACGGACGTAGGATACACTACGTTCTGGTCAGAATCCCTTGAGGCCGTTGACATTGAACATCGTGCTGCATGGCTGCTCGCTAAACAAGAGCGCAACCGGTTCCCGTTTGACACAAAAGCAATCGAAGAGTTGTACGTAGAGTTAGCTTCTCGCCGCTCTGAGTTGCTCCGTAAATTGACCGAAACGTTCGGCTCGTGGTATCAGcc                                                                                                                                                                                                                                                  <  1:282881/297‑1 (MQ=255)`  
                                                              `gagatgtgtataagagacaggtgGTAACTAAAGCTCTCCTTGAGAAGCTACTCTCTGACAAACATTACTTCCATCCTGAGATTGACTTTACGGACGTAGGATACACTACGTTCTGGTCAGAATCCCTTGAGGCCGTTGACATTGAACATCGTGCTGCATGGCTGCTCGCTAAACAAGAGCGCAACGGGTTCCCGTTTGACACAAAAGCAATCGAAGAGTTGTACGTAGAGTTAGCTTCTCGACGCTCTGAGTTGCTCCGTAAATTGACCGAAACGTTCGGCTCGTGGTATCAGCCTAAAgg                                                                                                                                                                                                                                            <  1:97888/281‑1 (MQ=255)`  
                                                              `ccTATAACGTTCAGGACGTTGTGGTAACTAAAGCTCTCCTTGAGAAGCTACTCTCTGACAAACATTACTTCCCTCCTGAGATTGACTTTACGGACGTAGGATACACTACGTTCTGGTCAGAATCCCTTGAGGCCGTTGACATTGAACATCGTGCTGCATGGCTGCTCGCTAAACAAGAGCGCAACGGGTTCCCGTTTGACACAAAAGCAATCGAAGAGTTGTACGTAGAGTTAGCTTCTCGCCGCTCTGAGTTGCTCCGTAAATTGACCGAAACGTTCGGCTCGTGGTATCAGCCTAAAgg                                                                                                                                                                                                                                            <  1:245655/300‑1 (MQ=255)`  
                                                              `aCTATAACGTTCAGGACGTTGTGGTAACTAAAGCTCTCCTTGAGAAGCTACTCTCTGACAAACATTACTTCCCTCCTGAGATTGACTTTACGGACGTAGGATACACTACGTTCTGGTCAGAATCCCTTGAGGCCGTTGACATTGAACATCGTGCTGCATGGCTGCTCGCTAAACAAGAGCGCAACGGGTTCCCGTTTGACACAAAAGCAATCGAAGAGTTGTACGTAGAGTTAGTTTCTCGCCGCTCTGAGTTGCTCCGTAAATTGACCGAAACGTTCGGCTCGTGGTATCAGCCTAAAgg                                                                                                                                                                                                                                            <  1:202912/301‑1 (MQ=255)`  
                                                              `aCTATAACGTTCAGGACGTTGTGGTAACTAAAGCTCTCCTTGAGAAGCTACTCTCTGACAAACATTACTTCCCTCCTGAGATTGACTTTACGGACGTAGGATACACTACGTTCTGGTCAGAATCCCTTGAGGCCGTTGACATTGAACATCGTGCTGCATGGCTGCTCGCTAAACAAGAGCGCAACGGGTTCCCGTTTGACACAAAAGCAATCGAAGAGTTGTACGTAGAGTTAGCTTCTCGCCGCTCTGAGTTGCTCCGTAAATTGACCGAAACGTTCGGCTAGTGGTATCAGCCTAAAgg                                                                                                                                                                                                                                            <  1:278685/301‑1 (MQ=255)`  
                                                              `aCTATAACGTTCAGGACGTGGTGGTAACTAAAGCTCTCCTTGAGAAGCTACTCTCTGGCAAACATTACTTCCCTCCTGAGATTGACTTTACGGACGTAGGATACACTACGTTCTGGTCAGAATCCCTTGAGGCCGTTGACATTGAACATCGTGCTGCATGGCTGCTCGCTAAACAAGAGCGCAACGGGTTCCCGTTTGAAACAAAAGCAATCGAAGAGTTGTACGTAGAGTTAGCTTCTCGACGCTCTGAGTTGCTCCGTAAATTGACCGAAACGTTCGGCTCGTGGTATCAGCCTAAAgg                                                                                                                                                                                                                                            <  1:159888/301‑1 (MQ=255)`  
                                                               `cTATAACGTTCAGGACGTTGTGGTAACTAAAGCTCTCCTTGAGAAGCTACTCTCTGACAAACATTACTTCCCTCCTGAGATTGACTTTACGGACGTAGGATACACTACGTTCTGGTCAGAATCCCTTGAGGCCGTTGACATTGAACATAGTGCTGCATGGCTGCTAGCTAAACAAGAGCGAAACGGGTTCCCGTTTGACACAAAAGCAATCGAAGAGTTGTACGTAGAGTTAGCTTCTCGCCGCTCTGAGTTGCTCCGTAAATTGACCGAAACGTTCGGCTCGTGGTATcctgtctcttat                                                                                                                                                                                                                                           >  1:7758/1‑290 (MQ=255)`  
                                                                  `tgtgtataagagacaggtgGTACCTAAAGCTCTCCTTGAGAAGCTACTCTCTGACAAACATTACTTCCCTCCTGAGATTGACTTTACGGACGTAGGATACACTACGTTCTGGTCAGAATCCCTTGAGGCCGTTGACATTGAACATCGTGCTGCATGGCTGCTCGCTAAACAAGAGCGCAACGGGTTCCCGTTTGACACAAAAGCAATCGAAGAGTTGTACGTAGAGTTAGCTTCTCGCCGCTCTGAGTTGCTCCGTAAATTGACCGAAACGTTCGGCTCGTGGTATCAGCCTAAAGGTGGc                                                                                                                                                                                                                                        <  1:335225/285‑1 (MQ=255)`  
                                                                    `aCGGTCAGGACGTTGTGGTAACTAAAGCTCTCCTTGAGAAGCTACTCTCTGACAAACATTACTTCCGTCCGGAGATTGACTTTACGGACGTAGGATACACTACGTTCTGGTCAGAATCCCTTGAGGCCGTTGACATTGAACATCGTGCTGCATGGCTGCTCGCTAAACAAGAGCGCAACGGGTTCCCGTTTGACACAAAAGCAATCGAAGAGTTGTACGTAGAGTTAGCTTCTCGCCGCTCTGAGTTGCTCCGTAAATTGACCGAAACGTTCGGCTCGTGGTATCAGCCTAAAGGTGGCAc                                                                                                                                                                                                                                      <  1:297476/301‑1 (MQ=255)`  
                                                                      `tttCAGGACGTTGTGGTAACTAAAGCTCTCCTTGAGAAGCTACTCTCTGACAAACATTACTTCCCTCCTGAGATTGACTTTACGGACGTAGGATACACTACGTTCTGGTCAGAATCCCTTGAGGCCGTTGACATTGAACATCGTGCTGCATGGCTGCTCGCTAAACAAGAGCGCAACGGGTTCCCGTTTGACACAAAAGCAATCGAAGAGTTGTACGTAGAGTTAGCTTCTCGCCGCTCTGAGTTGCTCCGTAAATTGACCGAAACGTTCGGCTCGTGGTATCAGCCTAAAGGTGGCACTg                                                                                                                                                                                                                                    <  1:263794/300‑1 (MQ=255)`  
                                                                      `gTTCAGGACGTTGTGGTAACTAAAGCTCTCCTTGAGAAGCTACTCTCTGACAAACATTACTTCCCTCCTGAGATTGACTTTACGGACGTAGGATACACTACGTTCTGGTCAGAATCCCTTGAGGCCGTTGACATTGACCATCGTGCTGCATGGCTGCTCGCTAAACAAGAGCGCAACGGGTTCCCGTTTGACACAAAAGCAATCGAAGAGTTGTACGTAGAGTTAGCTTCTCGCCGCTCTGAGTTGCTCCGTAAATTGACCGAAACGTTCGGCTCGTGGTATCAGCCTAAAggctgtctct                                                                                                                                                                                                                                    >  1:141871/1‑293 (MQ=255)`  
                                                                      `gTTCAGGACGTTGTGGTAACTAAAGCTCTCCTTGAGAAGCTACTCTCTGACAAACATTACTTCCCTCCTGAGATTGACTTTACGGACGTAGGATACACTACGTTCTGGTCAGAATCCCTTGAGGCCGTTGACATTGAACATCGTGCTGCATGGCTGCTCGCTAAACAAGAGCGCAACGGGTTCCCGTTTGACACAAAAGCAATCGAAGAGTTGTACGTAGAGTTAGCTTCTCGCCGCTCTGAGTTGCTCCGTAAATTGACCGAAACGTTCGGCTCGTGGTATCAGCCTAAAGGTGGCACTg                                                                                                                                                                                                                                    >  1:238102/1‑301 (MQ=255)`  
                                                                         `tataagagacaggTAACTAAAGCTCTCCTTGAGAAGCTACTCTCTGACAAACATTACTTCCCTCCTGAGATTGACTTTACGGACGTAGGATACACTACGTTCTGGTCAGAATCCCTTGAGGCCGTTGACATTGAACATCGTGCTGCATGGCTGCTCGCTAAACAAGATCGCAACGGGTTCCCGTTTGACACAAAAGCAATCGAATAGTTGTACGTAGAGTTAGCTTCTCGCCGCTCTGAGTTGCTCCGTAAATTGACCGAAACGTTCGGCTCGTGGTATCAGCCTAAAGGTGGCACTgaga                                                                                                                                                                                                                                 <  1:341440/290‑1 (MQ=255)`  
                                                                         `ggctcggagatgtgtataagagacagccTGGAGAAGCTACTCTCTGACAAACATTACTTCCCGCCTGAGATTGACTTTACGGACGTAGGATACACTACGTTCTGGTCAGAATCCCTTGAGGCCGTTGACATTGAACATCGTGCTGCATGGCTGCTCGCTAAACAAGAGCGCAACGGGTTCCCGTTTGACACAAAAGCAATCGAAGAGTTGTACGTAGAGTTAGCTTCTCGCCGCTCTGAGTTGCTCCGTAAATTGACCGAAACGTTCGGCTCGTGGTATCAGCCTAAAGGTGGCACTgaga                                                                                                                                                                                                                                 <  1:37962/275‑1 (MQ=255)`  
                                                                          `agtaCGTTGTGGTAACTAAAGCTCTCCTTGAGAAGCTACTCTCTGACAAACATTACTTCCCTCCTGAGATTGACTTTACGGACGTAGGATACACTACGTTCTGGTCAGAATCCCTTGAGGCCGTTGACATTGAACATCGTGCTGCATGGCTGCTCGCTAAACAAGAGCGCAACGGGTTCCCGTTTGACACAAAAGCAATCGACGAGTTGTACGTAGAGTTAGCTTCTCGCCGCTCTGAGTTGCTCCGTAAATTTACCGAAACGTTCGGCTCTTGGTATCAGCCTAAAGGTGGCACTGAGAt                                                                                                                                                                                                                                >  1:257757/4‑301 (MQ=255)`  
                                                                           `ggACGTTGTGGTAACTAAAGCTCTCCTTGAGAAGCTACTCTTTGACAAACATTACTTCCCTCCTGAGATTGACTTTACGTACGTAGTATACACTACGTTCTGGTCAGAATCCCTTGAGGCCGTTGACATTGAACATCTTGCTGCATTGCTGCTCGCTAAACAAGAGCGCAACGGGTTCACGTTTGACACAAAAGCAATCGAAGAGTTGTACGTAGAGTTAGCTTCTCGCCGCTCTGAGTTGCTCCGTAAATTGACCGAAACGTTCGGCTCGTGGTATCAGCCTAAAGGTGGCACTGAGATg                                                                                                                                                                                                                               <  1:48539/301‑1 (MQ=255)`  
                                                                           `ggACGTTGTGGTAACTAAAGCTCTCCTTGAGAAGCTACTCTCTGACAAACATTACTTCCCTCCTGAGATTGACTTTACGGACGTAGGATACACTACGTTCTGGTCAGAATCCCTTGAGGCCGTTGACATTGAACATCGTGCTGCATGGCTGCTCGCTAAACAAGAGCGCAACGGGTTCCCGTTTGACACAAAAGCAATCGAAGAGTTGTACGTAGAGTTAGCTTCTCGCCGCTCTGAGTTGCTCCGTAAATTGACCGAAACGTTCGGCTCGTGGTATCAGCCTAAAggctgtctcttatac                                                                                                                                                                                                                               >  1:305747/1‑288 (MQ=255)`  
                                                                             `aCGTTGTGGTAACTAAAGCTCTCCTTGAGAAGCTACTCTCTGACAAACATTACTTCCCTCCTAAGATGGACTTTACGGACGTAGGATACACTACGTTCTGGTCAGAATCCCTTGAGGCCGTTGACATTGAACATCGTGCTGCATGGCTGCTCGCTAAACAAGCGCGCGACGGGGTCCGGTTTGACACAAAAGAAATCGAAGAGTTGTACGTAGAGTTAGCTTCCCGCCGCGCTGAGTTGCTCCGTAACTTGACTGAACCGTTCGGCCCGTGGTAGTGGCCTAAAGGTgggagacgtctttt                                                                                                                                                                                                                             >  1:164298/1‑289 (MQ=255)`  
                                                                                   `tgGTAACTAAAGCTCTCATTGAGAAGCTACTCTCTGACAAACATTACTTCCCTCCTGAGATTGACTTTACGGACGTAGGATACACTACGTTCTGGTCAGAATCCCTTGAGGCCGTTGACATTGAACATCGTGCTGCATGGCTGCTCGCTAAACAAGAGCGCAACGGGTTCCCGTTTGACACAAAAGCAATCGAAGAGTTGTACGTAGAGTTAGCTTCTCGCCGCTCTGAGTTGCTCCGTAAATTGACCGAAACGTTCGGCTCGTGGTATCAGCCTAAAGGTGGCACTGAGATGTTCTGCCa                                                                                                                                                                                                                       <  1:41172/301‑1 (MQ=255)`  
                                                                                    `ggTAACTAAAGCTCTCCTTGAGAAGCTACTCTCTGACAAACATTACTTCCCTCCTGAGATTGACTTTACGGACGTAGGATACACTACGTTCTGGTCAGAATCCCTTGAGGCCGTTGACATTGAACATCGTGCTGCATGGCTGCTCGCTAAACAAGAGCGCAACGGGTTCCCGTTTGACACAAAAGCAATCGAAGAGTTGTACGTAGAGTTAGCTTCTCGCCGCTCTGAGTTGCTCCGTAAATTGACCGAAACGTTCGGCTCGTGGTATCAGCCTAAAGGTGGCACTGAGATGTTCTGcctg                                                                                                                                                                                                                      >  1:269906/1‑299 (MQ=255)`  
                                                                                       `cggagatgtgtataagagacaGCTACTCTCTGACAAACATTACTTCCCTCCTGAGATTGACTTTACGGACGTAGGATACACTACGTTCTGGTCAGAATCCCTTGAGGCCGTTGACATTGAACATCGTGCTGCATGGATGCTCGCTAAACAAGAGCGCAACGGGTTCCCGTTTGACACAAAAGCAATCGAAGAGTTGTACGTAGAGTTAGCTTCTCGCTGCTCTGAGTTGCTCCGTAAATTGACCGAAACGTTCGGCTCGTGGTATCAGCCTAAAGGTGGCACTGAGATGTTCTGCCATCcg                                                                                                                                                                                                                   <  1:50695/281‑1 (MQ=255)`  
                                                                                       `aaCTAAAGCTCTCCTTGAGAAGCTACTCTCTGACAAACATTACTTCCCTCCTGAGATTGACTTTACGGACGTAGGATACACTACGTTCTGGTCAGAATCCCTTGAGGCCGTTGACATTGAACATCGTGCTGCATGGCTGCTCGCTAAACAAGAGCGCAACGGGTTCCCGTTTGACACAAAAGCAATCGAAGAGTTGTACGTAGAGTTAGCTTCTCGCCGCTCTGAGTTGCTCCGTAAATTGACCGAAACGTTCGGCTCGTGGTATCAGCCTAAAggctgtctcttatacacatctccgagc                                                                                                                                                                                                                   >  1:148603/1‑276 (MQ=255)`  
                                                                                       `aaCTAAAGCTCTCCTTGAGAAGCTACTCTCTGACAAACATTACTTCCCTCCTGAGATTGACTTTACGGACGTAGGATACACTACGTTCTGGTCAGAATCCCTTGAGGCCGTTGACATTGAACATCGTGCTGCATGGCTGCTCGCTAAACAAGAGCGCAACGGGTTCCCGTTTGACACAAAAGCAATCGAAGAGTTGTACGTAGAGTTAGCTTCTCGCCACTCTGAGTTGCTCCGTAAATTGACCGAAACGTTCGGCTCGTGGTATCAGCCTAAAGGTGGCACTGAGATGTTCTGCCATCcg                                                                                                                                                                                                                   >  1:213946/1‑301 (MQ=255)`  
                                                                                         `cTAAAGCTCTCCTTGAGAAGCTACTCTCTGACAAACATTACTTCCCTCCTGAGATTGACTTTACGGACGTAGGATACACTACGTTCTGGTCAGAATCCCTTGAGGCCGTTGACATTGAACATCGTGCTGCATGGCTGCTCGCTAAACAAGAGCGCAACGGGTTCCCGTTTGACACAAAAGCAATCGAAGAGTTGTACGTAGAGTTAGCTTCTCGCCGCTCTGAGTTGCTCCGTAAATTGACCGAAACGTTCGGCTCGTGGTATCAGCCTAAAGGTGGCACTgagactgtctcttatacaca                                                                                                                                                                                                                 >  1:294526/1‑285 (MQ=255)`  
                                                                                            `aaGCTCTCATTGAGAAGCTACTCTCTGACAAACATTACTTCCCTCCTGAGATTGACTTTACGGACGTATGATACACTACGTTCTGGTCAGAATCCCTTGAGGCCGTCGACATTGAACATCGTTCTGCATGGCTGCTCGCTAAACAAGAGCGCAACGGGTTCCCGTTTGACACAAAAGCAATCGAAGAGTTGTACGTAGAGTTAGCTTCTCGCCGCTCTGAGTTGCTCCGTAAATTGACCGAAACGTTCGGCTCGTGGTATCAGCCTAAAGGTGGCACTGAGATGTTCTGCCATCAGCGAAc                                                                                                                                                                                                              <  1:316028/301‑1 (MQ=255)`  
                                                                                                   `ccTTGAGAAGCTACTCTCTGACAAACATTACTTCCCTCCTGAGATTGACTTTACGGACGTAGGATACACTACGTTCTGGTCAGAATCCCTTGAGGCCGTTGACATTGAACATCGTGCTGCATGGCTGCTCGCTAAACAAGAGCGCAACAGGTTCCCGTTTGACACAAAAGCAATCGAAGAGTTGTACGTAGAGTTAGCTTCTCGCCGCTCTGAGTTGCTCCGTAAATTGACCGAAACGTTCGGCTCGTGGTATCAGCCTAAAGGTGGCACTGAGATGTTCTGcctgtctcttatacacatc                                                                                                                                                                                                       >  1:56559/1‑284 (MQ=255)`  
                                                                                                     `cggagatgtgtataagagacagacaTTACTTCCCTCCTGAGATTGACTTTACGGACGTAGGATACACTACGTTCTGGTCAGAATCCCTTGAGGCCGTTGACATTGAACATCGTGCTGCATGGCTGCTCGCTAAACAAGAGCGCAACGGGTTCCCGTTTGACACAAAAGCAATCGAAGAGTTGTACGTAGAGTTAGCTTCTCGCCGCTCTGAGTTGCTCCGTAAATTGACCGAAACGTTCGGCTCGTGGTATCAGCCTAAAGGTGGCACTGAGATGTTCTGCCATCCGCGAACAGGTAAGcc                                                                                                                                                                                                     <  1:80523/284‑1 (MQ=255)`  
                                                                                                       `gagaAGCTACTCTCTGACAAACATTACTTCCCTCCTGAGATTGACTTTACGGACGTAGGATACACTACGTTCTGGTCAGAATCCCTGGAGGCCGTTGACATTGAACATCGTGCTGCATGGCTGCTCGCTAAACAAGAGCGCAACGGGTTCCCGTTTGACACAAAAGCAATCGAAGAGTTGTACGTAGAGTTAGCTTCTCGCCGCTCTGAGTTGCTCCGTAAATTGACCGAAACGTTCGGCTCGTGGTATCAGCCTAAAGGTGGCACTGAGATGTTCTGCCATCCGCGAACAGGTAAGCCAc                                                                                                                                                                                                   <  1:145242/301‑1 (MQ=255)`  
                                                                                                           `aGCTACTCTCTGACAACAATTACTTCCCTCCTGAGATGGACGTTACGGACGTAGGATACACTACGTTCTGGTCAGAATCCCTTGAGGCCGTTGACATTGAACATCGTGCTGCATGGCTGCTCGCTAAACAAGAGCGCAACGGGTTCCAGTTTGACACAAAAGCAATCGAAGAGTTGTACGTAGAGTTAGCTTCTCGCCGCTCTGAGTTGCTCCGTAAATTGACCGAAACGTTCGGCTCGTGGTATCAGCCTAAAGGTGGCACTGAGATGTTCTGCCATCCGCGAACAGGTAAGCCACTAcc                                                                                                                                                                                               <  1:46322/301‑1 (MQ=255)`  
                                                                                                            `gCTACTCTCTGACAAACATTACTTCCCTCCTGAGATTGACTTTACGGACGTAGGATACACTACGTTCTTGTCAGAATCCCTTGAGGCCGTTGACATTGAACATCGTGCTGCATGGCTGCTCGCTAAACAAGAGCGCAACGGGTTCCCGTTTGACACAAAAGCAATCGAAGAGTTGTACGTAGAGTTAGCTTCTCGCCGCTCTGAGTTGCTCCGTAAATTGACCGAAACGTTCGGCTCGTGGTATCAGCCTAAAGGTGGCACTGAGATGTTCTGCCATCCGCGAACCGGTAAGCCACTACCt                                                                                                                                                                                              >  1:182734/1‑301 (MQ=255)`  
                                                                                                               `aCTCTCTGACAAACATTACTTCCCTCCTGAGATTGACTTTACGGACGTAGGATACACTACGTTCTGGTCAGAATCCCTTGAGGCCGTTGACATTGAACATCGTGCTGCATGGCTGCTCGCTAAACAAGAGCGCAACGGGTTCCAGTTTGACACAAAAGCAATCGAAGAGTTGTACGTAGAGTTAGCTTCTCGCCGCTCTGAGTTGCTCCGTAAATTGACCGAAACGTTCGGCTCGTGGTATCAGCCTAAAGGTGGCACTGAGATGTTCTGCCATCCGCGAACAGGTAAGCCACTACCTaaa                                                                                                                                                                                           <  1:89350/301‑1 (MQ=255)`  
                                                                                                                `tataagagacagcATTACTTCCCTCCTGAGATTGACGTTACGGACGTAGGATACACTACGTTCTGGTCAGAATCCCGTGAGGCCGTTGACATTGAACATCGTGCTGCATGGCTGCTCGCTAAACAAGAGCGCAACGGGTTCCCGTTTGACACAAAAGCAATCGAAGAGTTGTACGTAGAGTTAGCTTCTCGCCGCTCTGAGTTGCTCCGTAAATTGACCGAAACGTTCGGCTCGTGGTATCAGCCTAAAGGTGGCACTGAGATGTTCTGCCATCCGCGAACAGGTAAGCCACTACCTAAAt                                                                                                                                                                                          <  1:20559/289‑1 (MQ=255)`  
                                                                                                                  `ctctGACAAACATTACTTCCCTCCTGAGATTGACTTTACGGACGTAGGATACACTACGTTCTGGTCAGAATCCCTTGAGGCCGTTGACATTGAACATCGTGCTGCATGGCTGCTCGCTAAACAAGAGCGCAACGGGTTCCAGTTTGACACAAAAGCAATCGAAGAGTTGTACGTAGAGTTAGCTTCTCGCCGCTCTGAGTTGCTCCGTAAATTGACCGAAACGTTAGGCTCGTGGTATCAGCCTAAAGGTGGCACTGAGATGTTCTGCCATCCGCGAACAGGTAAGCCACTACATAAATAc                                                                                                                                                                                        <  1:155103/301‑1 (MQ=255)`  
                                                                                                                   `tctGACAACCATTACTTCCCTCCTGAGATTGACTTTACGGACGTAGGATACACTACGTTCTGTTCAGAATCCCTTGAGGCCGTTGACATTGAACATCGTGCTGCATGGCTGCTCGCTAAACAAGAGCGCAACGGGTTCCCGTTTGACACAAAAGCAATCGAAGAGTTGTACGTAGAGTTAGCTTCTCGCCGCTCTGAGTTGCTCCGTAAATTGACCGAAACGTTCGTCTCGTGGTATCAGCCTAAAGGTGGCACTGAGATGTTCTGCCATCCGCGAACAGGTAAGCCACTACCTAAATAcc                                                                                                                                                                                       <  1:193775/301‑1 (MQ=255)`  
                                                                                                                    `ctGACAAACATTACTTCCCTCCTGAGATTGACTTTACGGACGTAGGATACACTACGTTCTGGTCAGAATCCCTTGAGGCCGTTGACATTGAACATCGTGCTGCATGGCTGCTCGCTAAACAAGAGCGCAACGGGTTCCCGTTTGACACAAAAGCAATCGAAGAGTTGTACGTAGAGTTAGCTTCTCGCCGCTCTGAGTTGCTCCGTAAATTGACCGAAACGTTCGGCTCGTGGTATCAGCCTAAAGGTGGCACTGAGATGTTCTGCCATcctgtctcttatacacatctcccagcccacga                                                                                                                                                                                      >  1:53582/1‑271 (MQ=255)`  
                                                                                                                     `tGACATACATTACTTCCCTCCTGGGATTGACTTTACGTACGTAGGATACACTACTTTCTGGTCAGAATCCCTTGTGGCCGTTGACATTGACCATCGTGCTGCATGGCTGCTCGCTAAACAAGAGCCCATCGGGTTCCCGTTTGACACAAAAGCAATCGAAGAGTTGTACGTAGAGTTAGCTTCTCGCCGCTCTGAGTTGCTCCGTAAATTGACCGAAACGTTCGTCTCGTGGTATCAGCCTAAAGGTGGCACTGAGATGTTCTGCCATCCGCGAACAGGTAAGCCACTACCTAAATACCCt                                                                                                                                                                                     <  1:100392/301‑1 (MQ=255)`  
                                                                                                                         `aaaCATTACTTCCCTCCTGAGATTGACTTTACGGACGTAGGATACACTACGTTCTGGTCAGAATCCCTTGAGGCCGTTGACATTGAACATCGTGCTGCATGGCTGCTCGCTAAACAAGAGCGCAACGTGTTCCCGTTTGACACAAAAGCAATCGAAGAGTTGTACGTAGAGTTAGCTTCTCGCCGCTCTGAGTTGCTCCGTAAATTGACCGAAACGTTCGGCTCGTGGTATCAGCCTAAAGGTGGCACTGAGATGTTCTGCCATCCGCGAACAGGTAAGCCACTACCTAAATACCCTCGCa                                                                                                                                                                                 <  1:134528/301‑1 (MQ=255)`  
                                                                                                                          `aaCATTACTTCCCTCCTGAGATTGACTTTACGGACGTAGGATACACTACGTTCTGGTCAGAATCCCTTGAGGCCGTTGACATTGAACATCGTGCTGCATGGCTGCTCGCTAAACAAGGGCGCAACGGGTTCCCGTTTGACACAAAAGCAATCGAAGAGTTGTACGTAGAGTTAGCTTCTCGCCGCTCTGAGTTGCTCCGTAAATTGACCGAAACGTTCGGCTCGTGGTATCAGCCTAAAGGTGGCACTGAGATGTTCTGCCATCCGCGAACAGGTAAGCCACTACCTAAATACCCTCGCAt                                                                                                                                                                                <  1:266884/301‑1 (MQ=255)`  
                                                                                                                            `cATTACTTCCCTCCTGATATTGACTTTACGGACGTAGGATACACTACGTTCTGGTCAGAATCCCTTGAGGCCGTTGACATTGAACATCGTGCTGCATGGCTGCTCGCTAAACAAGAGCGCAACGGGTTCCCGTTTGACACAAAAGCAATCGAAGAGTTGTACGTTGAGTTAGCTTCTCGCCGCTCTGAGTTGCTCCGTAAATTAACCGAAACGTTCGGCTCGTGGTATCAGCCTCACGGTGGCCCTGAGATGTTCTGCCATCCGCGAACAGGTACGCCACTACCTAAAtctgtctcttata                                                                                                                                                                              >  1:114584/1‑289 (MQ=255)`  
                                                                                                                            `cATTACTTCCCTCCTGAGATTGACTTTACGGACGTAGGATACACTACGTTCTGGTCAGAATCCCTTGAGGCCGTTGACATTGAACATCGTGCTGCATGGCTGCTCGCTAAACAAGAGCGCAACGGGTTCCCGTTTGACACAAAAGCAATCGAAGAGTTGTACGTAGAGTTAGCTTCTCGCCGCTCTGAGTTGCTCCGTAAATTGACCGAAACGTTCGGCTCGTGGTATCAGCCTAAAGGTGGCACTGAGATGTTCTGCCATCCGCGAACAGGTAAGCCAcctgtctcttatacacatctcc                                                                                                                                                                              >  1:255638/1‑280 (MQ=255)`  
                                                                                                                              `ttACTTCCCTCCTGAGATTGACTTTACGGACGTAGGATACACTACGTTCTGGTCAGAATCACTTGAGGCCGTTGACATTGAACATCGTGCTGCATGGCTGCTCGCTAAACAAGAGCGCAACGGGTTCCCGTTTGACACAAAAGCAATCGAAGAGTTGTACGTAGAGTTAGCTTCTCGCCGCTCTGAGTTGCTCCGTAAATTGACCGAAACGTTCGGCTCGTGGTATCAGCCTAAAGGTGGCACTGAGATGTTCTGCCATCCGCGAACAGGTAAGCCACTACCTAAATACCCTCGCATTAAg                                                                                                                                                                            >  1:69227/1‑301 (MQ=255)`  
                                                                                                                                `aCTTCCCTCCTGAGATTGACTTTACGGACGTAGGATACACTACGTTCTGGTCAGAATCCCTTGAGGCCGTTGACATTGAACATCGTGCTGCATGGCTGCTCGCTAAACAAGAGCGCAACGGGTTCCCGTTTGACACAAAAGCAATCGAAGAGTTGTACGTAGAGTTAGCTTCTCGCCGCTCTGAGTTTCTCCGTAAATTGACCGAAACGTTCTGCTCGTGGTATCAGCCTAAAGGTGGCACTGAGATGTTCTGCCATCCGCGAACAGGTAAGCCACTACCTAAATACCCTCGCATTAAGac                                                                                                                                                                          <  1:186047/301‑1 (MQ=255)`  
                                                                                                                                `aCTTCCCTCCTGAGATTGACTTTACGGACGTAGGATACACTACGTTCTGGTCAGAATCCCTTGAGGCCGTTGACATTGAACATCGTGCTGCATGGCTGCTCGCTAAACAAGAGCGCAACGGGTTCCCGTTTGACACAAAAGCAATCGAAGAGTTGTACGTAGAGTTAGCTTCTCGCCGCTCTGAGTTGCTCCGTAAATTGACCGACACGTTCGGCTCGTGGTATCAGCCTAAAGGTGGCACTGAGATGTTCTGCCATCCGCGAACAGGTAAGCCACTACCTAAATACCCTCGCATTAAgcc                                                                                                                                                                          >  1:292289/1‑299 (MQ=255)`  
                                                                                                                                  `ttCCCTCCTGAGATTGACTTTACGGACGTAGGATACACTACGTTCTGGTCAGAATCCCTTGAGGCCGTTTACATTGAACATCGTGCTGCATGGCTGCTCGCTAAACAAGAGCGCAACGGGTTCCCGTTTGACACAAAAGCAATCGAAGAGTTGTACGTAGAGTTAGCTTCTCGCCGCTCTGAGTTGCTCCGTAAATTGACCGAAACGTTCGGCTCGTGGTATCAGCCTAAAGGTGGCACTGAGATGTTCTGCCATCCGCGAACAGGTAAGCCACTACCTAAATACCCTCGCATTAAGacac                                                                                                                                                                        <  1:95348/301‑1 (MQ=255)`  
                                                                                                                                        `gacaggaTTGACTTTACGGACGTAGGATACACTACGTTCTGGTCAGAATCCCTTGAGGCCGTTGACATTGAACATCGTGCTGCATGGCTGCTCGCTAAACAAGAGCGCAACGGGTTCCCGTTTGACACAAAAGCAATCGAAGAGTTGTACGTAGAGTTAGCTTCTCGCCGCTCTGAGTTGCTCCGTAAATTGACCGAAACGTTCGGCTCGTGGTATCAGCCTAAAGGTGGCACTGAGATGTTCTGCCATCCGCGAACAGGTAAGCCACTACCTAAATACCATCGCATTAAGACACCTAAAg                                                                                                                                                                  <  1:209835/296‑1 (MQ=255)`  
                                                                                                                                        `cctGAGATTGACTTTACGGACGTAGTATCCACTACGTTCTGGTCAGAATCCCTTGAGGCCGTTGACATTGAACATCGTGCTGCATGGCTGCTCGCTAAACAAGAGCGCAACGGGTTCCCGTTTGACACAAAAGCAATCGAAGAGTTGTACGTAGAGTTAGCTTCTCGCCGCTCTGAGTTGCTCCGTAAATTGACCGAAACGTTCGGCTCGTGGTATCAGCCTAAAGGTGGCACTGAGATGTTCTGCCATCCGCGAACAGGTAAGCCACTACCTAAATACCCTCGCATTAAGACACCTAAAg                                                                                                                                                                  <  1:250340/301‑1 (MQ=255)`  
                                                                                                                                        `cctGAGATTGACTTTACGGACGTAGGATACACTACGTTCTGGTCAGAATCCCTTGAGGCCGTTGACATTGAACAACGTGCTGCATGGCTGCTCGCTAAACAAGCGCGCAACGGGTTCCCGTTTGACACAAAAGCAATCGAAGAGTTGTACGTAGAGTTAGCTTCTCGCCGCTCTGAGTTGCTCCGTAAATTGACCGAAACGTTCGGCTCGTGGTATCAGCCTAAAGGTGGAACTGAGATGTTCTGCCATCCGCGAACAGGTAAGCCACTACCTAAATACCCTCGCATTAAGACACCTAAAg                                                                                                                                                                  <  1:166819/301‑1 (MQ=255)`  
                                                                                                                                                   `cTTTACGGACGTAGGATACAATACGTTCTGGGCAGAATCCCTTGAGGCCGTTGACATTGAACATCGTGCTGCATGGCTGCTCGCTAAACAAGAGCGCAACGGGTTCCCGTTTGACACAAAAGCAATCGAAGAGTTGTACGTAGAGTTAGCTTCTCGCCGCTCTGAGTTGCTCCGTAAATTGACCGAAACGTTCGGCTCGTGGTATCAGCCTAAATGTGGCACTGAGATGTTCTGCCATCCGCGAACAGGTAAGCCACTACCTAAATACCCTCGCATTAAGACACCTAAAGTTGGTGGTATc                                                                                                                                                       <  1:301978/301‑1 (MQ=255)`  
                                                                                                                                                         `ggACGTAGGATACACTACGTTCTGGTCAGAATCCCTTGAGGCCGTTGACATTGAACATCGTGCTGCATGGCTGCTCGCTAAACAAGAGTGCAACGGGTTCCCGTTTGACACAAAAGCAATCGAAGAGTTGTACGTAGAGTTAGCTTCTCGCCGCTCTGAGTTGCTCCGTAAATTTACCGAAACGTTCGGCTCGTGGTATCAGCCTAAAGGTGGCACTGAGATGTTCTGCCATCCGCGAACAGGTAAGCCACTACCTAAATACCCTCGCATTAAGACACCTAAAGTTGGTGGTATCTTTaag                                                                                                                                                 <  1:229582/301‑1 (MQ=255)`  
                                                                                                                                                           `ccGTAGGATACACTACGTTCTGGTCAGAATCCCTTGCGGCCGTTGACATTGAACATCGTGCTGCATGGCTGCTCGCTAAACAAGAGCGCAACGGGTTCCCGTTTGACACAAAAGCAATCGAAGAGTTGTACGTAGAGTTAGCTTCTCGCCGCTCTGAGTTGCTCCGTAAATTGACCGAAACGTTCGGCTCGTGGTATCAGCCTAAAGGTGGCACTGAGATGTTCTGCCATCCGCGAACAGGTAAGCCACTACCTAAATACCCTCGCATTAAGACACCTAAAGTTGGTGGTATCTTTaagaa                                                                                                                                               <  1:235371/300‑1 (MQ=255)`  
                                                                                                                                                               `aGGATACACTACGTTCTGGTCAGAATCCCTTGAGGCCGTTGACATTGAACATCGTGCTGCATGGCTGCTCGCTAAACAAGAGCGCAACGGGTTCCCGTTTGACACAAAAGCAATCGAAGAGTTGTACGTAGAGTTAGCTTCTCGCCGCTCTGAGTTGCTCCGTAAATTGACCGAAACGTTCGGCTCGTGGTATCAGCCTAAAGGTGGCACTGAGATGTTCTGCCATCCGCGAACAGGTAAGCCACTACCTAAATACCCTCGCATTAAGACACCTAAAGTTGGTGGTATCTTTAAGAAGCCt                                                                                                                                           >  1:289396/1‑301 (MQ=255)`  
                                                                                                                                                                 `gATACACTACGTTCTGGTCAGAATCCCTTGAGGCCGTTGACATTGAACATCGTGCTGCATGGCTGCTCGCTAAACAAGAGCGCAACGGGGTCCCGTTTGACACAAAAGCAATCGAATAGTTGTACGTAGAGTTAGCTTCTCGCCGCTCTGAGTTGCTCCGTAAATTGACCGAAACGTTCGGCTCGTGGTATCAGCCTAAAGGTGGCACTGAGATGTTCTGCCATCCGCGAACAGGTAAGCAACTACCTAAATACCCTCGCATTAAGACACATAAAGTTGGTGGTATCTTTaagctgtctct                                                                                                                                         >  1:172479/1‑293 (MQ=255)`  
                                                                                                                                                                     `cacTACGTTCTGGTCAGAATCCCTTGAGGCCGTTGACATTGAACATCGTGCTGCATGGCTGCTCGCTAACCAAGAGCGCAACGGGTTCCCGTTTGACACAAAAGCAATCGAAGAGTTGTACGTAGAGTTAGCTTCTCGCCGCTCTGAGTTGCTCCGTAAATTGACCGAAACGTTCGGCTCGTGGTATCAGCCTAAAGGTGGCACTGAGATGTTCTGCCATCCGCGAACAGGTAAGCCACTACCTAAATACCCTCGCATTAAGACACCTAAAGTTGGTGGTATCTTTAAGAAGCCTAAGAAc                                                                                                                                     <  1:285584/301‑1 (MQ=255)`  
                                                                                                                                                                     `cacTACGTTCTGGTCAGAATCCCTTGAGGCCGTTGACATTGAACATCGTGCTGCATGGCTGCTCGCTAAACAAGAGCGCAACGGGTTCCCGTTTGACACAAAAGCAATCGAAGAGTTGTACGTAGAGTTAGCTTCTCGCCGCTCTGAGTTGCTCCGTAAATTGACCGAAACGTTCGGCTCGTGGTATCAGCCTAAAGGTGGCACTGAGATGTTCTGCCATCCGCGAACAGGTAAGCCACTACCTAAATACCCTCGCATTAAGACACCTAAAGTTGGTGGTATCTTTAAGAAGCCTAAGaaa                                                                                                                                     >  1:91739/1‑300 (MQ=255)`  
                                                                                                                                                                     `cacTACGTTCTGGTCAGAATCCCTTGAGGCCGTTGACATTGAACATCGTGCTGCATGGCTGCTCGCTAAACAAGAGCGCAACGGGTTCCCGTTTGACACAAAAGCAATCGAAGAGTTGTACGTAGAGTTAGCTTCTCGCCGCTCTGAGTTGCTACGTAAATTGACCGAAACGGTCGGCTCGTGGTATCAGCCTAAAGGTGGCACTGAGATGTTCTGCCATCCGCGAACAGGTAAGCCACTACCTAAATACCCTCGCATTAAGACACCTAAAGTTGGTGGTATCTTTAAGAAGCCTAAGAAc                                                                                                                                     <  1:223231/301‑1 (MQ=255)`  
                                                                                                                                                                       `cTACGTTCTGGTCAGAATCCCTTGAGGCCGTTGACATTGAACATCGTGCTGCATGGCTGCTCGCTAAACAAGAGCGCAACGGGTTCCCGTTTGACACAAAAGCAATCGAAGAGTTGTACGTAGAGTTAGCTTCTCGCCGCTCTGAGTTGCTCCGTAAATTGACCGAAACGTTCGGCTCGTGGTATCAGCCTAAAGGTGGCACTGAGATGTTCTGCCATCCGCGAACAGGTAAGCCACTACCTAAATACCCTCGCATTAAGACACCTAAAGTTGGTGGTATCTTTAAGAAGCCTAAGAACaa                                                                                                                                   >  1:318462/1‑301 (MQ=255)`  
                                                                                                                                                                                `ggTCAGAATCCCTTGAGGCCGTTGACATTGAACATCGTGCTGCATGGCTGCTCGCTAAACAAGAGCGCAACGGGTTCCCGTTTGACACAAAAGCAATCGAAGAGTTGTACGTAGAGTTAGCTTCTCGCCGCTCTGAGTTGCTCCGTAAATTGACCGAAACGTTCGGCTCGTGGTATCAGCCTAAAGGTGGCACTGAGATGTTCTGCCATCCGCGAACAGGTAAGCCACTACCTAAATACCCTCGCATTAAGACACCTAAAGTTGGTGGTATCTTTAAGAAGCCTAAGAACAAGGcctgtct                                                                                                                          >  1:59876/1‑295 (MQ=255)`  
                                                                                                                                                                                `ggTCAGAATCCCTTGAGGCCGTTGACATTGAACATCGTGCTGCATGGCTGCTCGCTAAACAAGAGCGCAACGGGTTCCCGTTTGACACAAAAGCAATCGAAGAGTTGTACGTAGAGTTAGCTTCTCGCCGCTCTGAGTTGCTCCGTAAATTGACCGAAACGTTCGGCTCGTGGTATCAGCCTAAAGGTGGCACTGAGATGTTCTGCCATCCGCGAACAGGTAAGCCACTACCTAAATACCCTCACATTAAGACACCTAAAGTTGGTGGTATCTTTAAGAAGCCTAAGAACAAGGCACAGCg                                                                                                                          >  1:85613/1‑301 (MQ=255)`  
                                                                                                                                                                                     `gAATCCCTTGAGGCCGTTGACATTGAACATCGTGCTGCATGGCTGCTCGCTAAACAAGAGCGCAACGGGTTCCCGTTTGACACAAAAGCAATCGAAGAGTTGTACGTAGAGTTAGGTTCTCGCCGCTCTGAGTTGCTCCGTAAATTGACCGAAACGTTCGGCTCGTGGTATCAGCCTAAAGGTGGCACTGAGATGTTCTGCCATACGCGAACAGGTAAGCAACTACCTAAATACCCTCGCATTAAGACAACTAAAGTTGGTGGTATCTTTaagactgtctcttatacacatctccgagccc                                                                                                                     >  1:162008/1‑274 (MQ=255)`  
                                                                                                                                                                                       `aTCCCTTGAGGCCGTTGACATTGAACATCGTGCTGCATGGCTGCTCGCTAAACAAGAGCGCAACGGGTTCCCGTTTGACACAAAAGCAATCGAAGAGTTGTACGTAGAGTTAGCTTCTCGCCGCTCTGAGTTGCTACGTAAATTGACCGAAACGTTCGGCTCGTGGTATCAGCCTAAAGGTGGCACTGAGATGTTCTGCCATCCGCGAACAGGTAAGCCACTACCTAAATACCCTCGCATTAAGACACCTAAAGTTGGTGGTATCTTTAAGAAGCCTAAGAACAAGGCACAGCGAGAAGGc                                                                                                                   >  1:178250/1‑301 (MQ=255)`  
                                                                                                                                                                                                   `cGTTGACATTGAACATCGTGCTGCATGGCTGCTCGCTAAACAAGAGCGCAACGGGTTCCCGTTTGACACAAAAGTAATCGAAGAGTTGTACGTAGAGTTAGCTTCTCGCCGCACCGAGTTGCTCCGTAAAGTGAACGAAACGTTCGGCCTGTGGGATCAGCCTAAAGGTGGCACTGAGATGTTCGGCCATCCGCGAAAAGGTAAGCCACTACCAAAATAACGTCGCATTAAGAAACCGAAAGTTGGTGGTAACTTTAAGAAGCCAAAGAACAAGGCACAGCGAGAGGGCAGTGAGACtggg                                                                                                       >  1:248417/1‑298 (MQ=255)`  
                                                                                                                                                                                                       `gACATTGAACATCGTGCTGCATGGCTGCTCGCTAAACAAGAGCGCAACGGGTTCCCGTTTGACACAAAAGCAATCGAAGAGTTGTACGTAGAGTTAGCTTCTCGCCGCTCTGAGTTGCTCCGTAAATTGACCGAAACGTTCGGCTCGTGGTATCAGCCTAAAGGTGGCACTGAGATGTTCTGCCATCCGCGAACAGGTAAGCCACTACCTAAATACCCTCGCATTAAGACACCTAAAGTTGGTGGTATCTTTAAGAAGCCTAAGAACAAGGCACAGCGAGAAGGCCGTGAGCCTTGCGAAc                                                                                                   >  1:30970/1‑301 (MQ=255)`  
                                                                                                                                                                                                        `aCATTGAACATCGTGCTGCATGGCTGCTCGCTAAACAAGAGCGCAACGGGTTCCCGTTTGACACAAAAGCAATCGAAGAGTTGTACGTAGAGTTAGCTTCTCGCCGCTCTGAGTTGCTCCGTAAATTGACCGAAACGTTCGGCTCGTGGTATCAGCCTAAAGGTGGCACTGAGATGTTCTGCCATCCGCGAACAGGTAAGCCACTACCTAAATACCCTCTCATTAAGACACCTAAAGTTGGTGGTATCTTTAAGAAGCCTAAGAACAAGGCACAGCGAGAATGCCGTGAGCCTTGCGAACt                                                                                                  >  1:252353/1‑301 (MQ=255)`  
                                                                                                                                                                                                         `cATTGAACATCGTGCTGCATGGCTGCTCGCTAAACAAGAGCGCAACGGGTTCCCGTTTGACACAAAAGCAATCGAAGAGTTGTACGTAGAGTTAGCTTCTCGCCGCTCTGAGTTGCTCCGTAATTTGACCGAAACGTTCGGCTCGTGGTATCAGCCT‑AAGGTGGCACTGAGATGTTCTGCCATCCGCGAACAGGTAAGCCACTACCTAAATACCCTCGCATTAAGACACCTAAAGTTGGTGGTATCTTTAAGAAGCCTAAGAACAAGGCACAGCGAGAAGGCCGTGAGCCTTGCGAACTTg                                                                                                >  1:11097/1‑301 (MQ=255)`  
                                                                                                                                                                                                                `cATCGTGGTGCTTGGCTGCTCGCAAAACAAGAGCCAAACGGTTTCCCGTTTGACAAAAAAGCAATGGAAGATTTTAACTTGGATTTAGCTTCCCGCCGTTCTGAGTTGTTCCGTAAATGGACCGAAACGTTCGGCTCGTGTAATCAGCTTAAAGTTGGCACTGAGAGTTTCTGCCATCCGCGACCAGGTAAGCCACTACCT‑AATACCCTCGCATTAAGACACCTAAAGTTGGTGGTATCTTTAAGAAGCCTAAGAACAAGGCACAGCGAGAAGGCCGTGAGCCTTGCGAACTTGATACCcg                                                                                         <  1:175410/301‑1 (MQ=255)`  
                                                                                                                                                                                                                     `tgctgcATGGCTGCTCGCTAAACAAGAGCGCAACGGGTTCCCGTTTGACACAAAAGCAATTGAAGAGTTGTACGTAGAGTTAGCTTCTCGCCGCTCTGAGTTGCTCCGTAAATTGACCGAAACGTTCGGCTCGTGGTATCAGCCTAAAGGTGGCACTGAGATGTTCTGCCATCCGCGAACAGGTAAGCCACTACCTAAATACCCTCGCATTAAGACACCTAAAGTTGGTGGTATCTTTAAGAAGCCTAAGAACAAGGCACAGCGAGAAGGCCGTGAGCCTTGCGAACTTGATACCCGCGAg                                                                                     >  1:151668/1‑301 (MQ=255)`  
                                                                                                                                                                                                                     `tgctgcATGGCTGCTCGCTAAACAAGAGCGCAACGGGTTCCCGTTTGACACAAAAGCAATCGAAGAGTTGTACGTAGAGTTAGCTTCTCGCCGCTCTGAGTTGCTCCGTAAATTGACCGAAACGTTCGGCTCGTGGTATCAGCCTAAAGGTGGCACTGAGATGTTCTGCCATCCGCGAACAGGTAAGCCACTACCTAAATACCCTCGCATTAAGACACCTAAAGTTGGTGGTATCTTTAAGAAGCCTAAGAACAAGGCACAGCGAGAAGGCCGTGAGCCTTGCGAACTTGATACCCGCGAg                                                                                     <  1:131751/301‑1 (MQ=255)`  
                                                                                                                                                                                                                          `cATGGCTGCTCGCTAAACAAGAGCGCAACGGGTTCCCGTTTGACACAAAAGCAATCGAAGAGTTGTACGTAGAGTTAGCTTCTCGCCGCTCTGAGTTGCTCCGTAAATTGACCGAAACGTTCGGCTCGTGGTATCAGCCTAAAGGTGGCACTGAGATGTTCTGCCATCCGCGAACAGGTAAGCCACTACCTAAATACCCTCGCATTAAGACACCTAAAGTTGGTGGTATCTTTAAGAAGCCTAAGAACAAGGCACAGCGAGAAGGCCGTGAGCCTTGCGAACTTGATACCCGCGAGTACGt                                                                                <  1:326131/301‑1 (MQ=255)`  
                                                                                                                                                                                                                                    `cGCTAAACAAGAGCGCAACGGGTTCCCGTTTGACACAAAAGCAATCGAAGAGTTGTACGTAGAGTTAGCTTCTCGCCGCTCTGAGTTGCTCCGTAAATTGACCGAAACGTTCGGCTCGTGGTATCAGCCTAAAGGTGGCACTGAGATGTTCTGCCATCCGCGAACAGGTAAGCCACTACCTAAATACCCTCGCATTAAGACACCTAAAGTTGGTGGTATCTTTAAGAAGCCTAAGAACAAGGCACAGCGAGAAGGCCGTGAGCCTTGCGAACTTGATACCCGCGAGTACGTTGCTGGTGCt                                                                      >  1:240288/1‑301 (MQ=255)`  
                                                                                                                                                                                                                                    `cGCTAAACAAGAGCGCAACGGGTTCCCGTTTGACACAAAAGCAATCGAAGAGTTGTACGTAGAGTTAGCTTCTCGCCGCTCTGAGTTGCTCCGTAAATTGACCGAAACGTTCGGCTCGTGGTATCAGCCTAAAGGTGGCACTGAGATGTTCTGCCATACGCGAACAGGTAAGCCACTACCTAAATACCCTCGCATTAAGACACCTAAAGTTGGTGGTATCTTTAAGAAGCCTAAGAACAAGGCACAGCGAGAAGGCCGTTAGCCTTGCGAACTTGATACCCGCGAGTACGTTGCTGGTGcc                                                                      >  1:313168/1‑300 (MQ=255)`  
                                                                                                                                                                                                                                     `gCTAAACAAGAGCGCAACGGGTTCCCGTTTGACACAAAAGCAATCGAAGAGTTGTACGTAGAGTTAGCTTCTCGCCGCTCTGAGTTGCTCCGTAAATTGACCGAAACGTTCGGCTCGTGGTATCAGCCTAAAGGTGGCACTGAGATGTTCTGCCATCCGCGAACAGGTAAGCCACTACCTAAATACCCTCGCATTAAGACACCTAAAGTTTGTGGTATCTTTAAGAAGCCTAAGAACAAGGCACAGCGAGAAGGCCGTGAGCCTTGCGAACTTGATACCCGCGAGTACGTTGCTGGTGCTc                                                                     <  1:103645/301‑1 (MQ=255)`  
                                                                                                                                                                                                                                      `cTAAACAAGAGCGCAACGGGTTCCCGTTTGACACAAAAGCAATCGAAGAGTTGTACGTAGAGTTAGCTTCTCGCCGCTCTGAGTTGCTCCGTAAATTGACCGAAACGTTCGGCTCGTGGTATCAGCCTAAAGGTGGCACTGAGATGTTCTGCCATCCGCGAACAGGTAAGCCACTACCTAAATACCCTCGCATTAAGACACCTAAAGTTGGTGGTATCTTTAAGAAGCCTAAGAACAAGGCACAGCGAGAAGGCCGTGAGCCTTGCGAACTTGATACCCGCTAGTACGTTGCTGGTGCTcc                                                                    >  1:338493/1‑301 (MQ=255)`  
                                                                                                                                                                                                                                                `gcgcAACGGGTTCCCGTTTGACACAAAAGCAATCGAAGAGTTGTACGTAGAGTTAGCTTCTCGCCGCTCTGAGTTGCTCCGTAAATTGACCGAAACGTTCGGCTCGTGGTATCAGCCTAAAGGTGGCACTGAGATGTTCTGCCATCCGCGAACAGGTAAGCCACTACCTAAATACCCTCGCATTAAGACACCTAAAGTTGGTGGTATCTTTAAGAAGCCTAAGAACAAGGCACAGCGAGAAGGCCGTGAGCCTTGCGAACTTGATACCCGCGAGTACGTTGCTGGTGCTCCTTACACCCCa                                                          >  1:106609/1‑301 (MQ=255)`  
                                                                                                                                                                                                                                                     `aCGGGTTCCCGTTTGACACAAAAGCAATCGAAGAGTTGTACGTAGAGTTAGCTTCTCGCCGCTCTGAGTTGCTCCGTAAATTGACCGAAACGTTCGGCTCGTGGTATCAGCCTAAAGTTGGCACTGAGATGTTCTGCCATCCGCGAACAGGTAAGCCACTACCTAAATACCCTCGCATTAAGACACCTAAAGTTGGTGGTATCTTTAAGAAGCCTAAGAACAAGGCACAGCGAGAAGGCCGTGAGCCTTGCGAACTTGATACCCGCGAGTACGTTGCTGGTGCTCCTTACACCCCAGTTGa                                                     >  1:217886/1‑301 (MQ=255)`  
                                                                                                                                                                                                                                                         `gTTCCCGTTTGTCACAAAAGCAATCGAAGAGTTGTACGTAGAGTTAGCTTCTCGCCGCTCTGAGTTGCTCCGTAAATTGACCGAAACGTTCGGCTCGTGGTATCAGCCTAAAGGTGGCACTGAGATGTTCTGCCATCCGCGAACAGGTAAGCCACTACCTAAATACCCTCGCATTAAGACACCTAAAGTTGGTGATATCTTTAAGAAGCCTAAGAACAAGGCACAGCGAGAAGGCCGTGAGCCTTGCGAACTTGATACCCGCGAGTACGTTGCTGGTGCTCCTTACACCCCAGTTGAACAt                                                 <  1:331376/301‑1 (MQ=255)`  
                                                                                                                                                                                                                                                         `gTTCCCGTTTGACACAAAAGCAATCGAAGAGTTGTACGTAGAGTTAGCTTCTCGCAGCTCTGAGTTGCTCCGTAAATTGACCGAAACGTTCGGCTCGTGGTATCAGCCTAAAGGTGGCACTGAGATGTTCTGCCATCCGCGAACAGGTAAGCCACTACCTAAATACCCTCGCATTAAGACACCTAAAGTTGGTGGTATCTTTAAGAAGCCTAAGAACAAGGCACAGCAAGAAGGCCGTGAGCCTTGCGAACTTGATACCCGCGAGTACGTTGCTGGTGCTCCTTacctgtctcttatacac                                                 >  1:13824/1‑286 (MQ=255)`  
                                                                                                                                                                                                                                                                     `taagagacagctCGAAGAGTTGTACGTAGAGTTAGCGTCTGGCCGCTCTGAGTTGCGCCGTAAATTGACCGAAACGTTCGGCTCGTGGTATCAGCCTAAAGGTGGAAATGAGATGGTCTGCCAGCCGCGAACAGGTAAGCCTCTACATAAATACCCTCGCATTAAGACACCTAAAGTGGGTGGTATCTTTAAGAAGCATAAGAACAAGGCACAGCGAGAAGGCCGTGAGCCTTGCGAACTTGATACCCGCGAGTACGTTGCTGGTGCTCCTTACACCCCAGTTGAACATGTTGTGTTTaat                                     <  1:189565/290‑2 (MQ=255)`  
                                                                                                                                                                                                                                                                     `aacaAAAGCAATCGAAGAGTTGTACGTAGAGTTAGCTTCTCGCCGCTCTGAGTTGCTCCGTAAATTGACCGAAACGTTCGTCTCGTGGTATCAGCCTAAAGGTGGCACTGAGATGTTCTGCCATCCGCGAACAGGTAAGCCACTACCTAAATACCCTCGCATTAAGACACCTAAAGTTGGTGGTATCTTTAAGAAGCCTAAGAACAAGGCACAGCGAGAAGGCCGTGAGCCTTGCGAACTTGATACCCGCGAGTACGTTGCTGGTGCTCCTTACACCCCAGTTGAACATGTTGTGTTTAAc                                     <  1:275670/300‑1 (MQ=255)`  
                                                                                                                                                                                                                                                                       `tgtgtataatagacaggTTGTATGTAGAGTTAGCTTCGCGCCGCTCTGAGTTGCTCCGTAAATTGACCGAAACGTTCGGCTCGTGGTATCAGCCTAAAGGTGGCACTGAGATGTTCTGCCATCCGCGAACAGGTAAGCCACTACCTAAATACCCTCGCATTAAGACACCTAAAGTTGGTGGTATCTTTAAGAAGCCTAAGAACAAGGCACAGCGAGAAGGCCGTGAGCCTTGCGAACTTGATACCCGCGAGTACGTTGCTGGTGCTCCTTACACCCCAGTTGAACATGTTGTGTTTAAcct                                   <  1:232929/285‑2 (MQ=255)`  
                                                                                                                                                                                                                                                                       `cgaaggCAAGCGAAGATTGGTACGTAGAGTTAGCGTCTCTCCGCTCTGAGTTGCTCCGTAAATTGGCCGGAACGTTCGGCTCGTAGTATCAGACTAAAGGTAGCACTGAGATGTTCTGCCATCCGCGAACAGGTAAGCCACTACCTAAATACCCTCGCATAAAGACACCTAAAGTTGATGGTATCTTTAAGAAGCCTAAGAACAAGGCACAGCGAGAAGGCCGTGAGCCTTGCGAACTTGATACCCGCGAGTACGTTGCTGGTGCTCCTTACACCCCAGTTGAACATGTTGTGTTTAAacc                                   <  1:35882/296‑1 (MQ=255)`  
                                                                                                                                                                                                                                                                               `aTCGAAGAGTTGTACGTAGAGTTAGCTTCTCGCCGCTCTGAGTTGCTCCGTAAATTGACCGAAACGTTCGGCTCGTGGTATCAGCCTAAAGGTGGCACTGAGATGTTCTGCCATCCGCGAACAGGTAAGCCACTACCTAAATACCCTCGCATTAAGACACCTAAAGTTGGTGGTATCTTTAAGAAGCCTAAGAACAAGGCACAGCGAGAAGGCCGTGAGCCTTGCTAACTTGATACCCGCGAGTACGTTGCTGGTGCTCCTTACACCCCCGTTGAACATGTTGTGTTTAACTCTTCGtctt                           >  1:196938/1‑300 (MQ=255)`  
                                                                                                                                                                                                                                                                               `aTCGAAGAGTTGTACGTAGAGTTAGCTTCTCGCCGCTCTGAGTTGCTCCGTAAATTGACCGAAACGTTCGGCTCGTGGTATCAGCCTAAAGGTGGCACTGAGATGTTCTGCCATCCGCGAACAGGTAAGCCACTACCTAAATACCCTCGCATTAAGACACCTAAAGTTGGTGGTATCTTTAAGAAGCCTAAGAACAAGGCACAGCGAGAAGGCCGTGAGCCTTGCGAACTTGATACCCGCGAGTACGTTGCTGGTGCTCCTTACACCCCAGTTGAACATGTTGTGTTTAACCCTTCGtctg                           >  1:15776/1‑300 (MQ=255)`  
                                                                                                                                                                                                                                                                               `aTCGAAGAGTTGTACGTAGAGTTAGCTTCTCGCCGCTCTGAGTTGCTCCGTAAATTGACCGAAACATTCGGCTCGTGGTATCAGCCTAAAGGTGGCACTGAGATGTTCTGCCATCAGCGAACAGGTAAGCCACTACCTAAATACCCTCGCATTAAGACACCTAAAGTTGGTGGTATCTTTAAGAAGCCTAAGAACAAGGCACAGCGAGAAGGCCGTGAGCCTTGCGAACTTGATACCCGCGAGTACGTTGCTGGTGCTCCTTACACACCAGTTGAACATGTTGTGTTTAACCCTTCGtctc                           >  1:169510/1‑301 (MQ=255)`  
                                                                                                                                                                                                                                                                                     `gagTTGTACGTAGAGTTAGCTTCTCGCCGCTCTGAGTTGCTCCGTAAATTGACCGAAACGTTCGGCTCGTGGTATCAGCCTAAAGGTGGCACTGAGATGTTCTGCCATCCGCGAACAGGTAAGCCACTACCTAAATACCCTCGCATTAAGACACCTAAAGTTGGTGGTATCTTTAAGAAGCCTAAGAACAAGGCACAGCGAGAAGGCCGTGAGCCTTGCGAACTTGATACCCGCGAGTACGTTGCTGGTGCTCCTTACACCCCAGTTGAAAATGTTGTGTTTAAcccctggctcttataca                     >  1:65810/1‑287 (MQ=255)`  
                                                                                                                                                                                                                                                                                     `gagTTGTACGTAGAGTTAGCTTCTCGCCGCGCTGAGGTGCTCCGTAAATTGACCGAAACGTTCGGCTCGTGGTATCAGCCTAAAGGTGGCACTGAGATGTTCTGCCATCCGCGAACAGGTAAGCCAATACCTAAATACCCTCGCATTAAGACACCTAAAGTTGGTGGTATCTTTAAGAAGCCTAAGAACAAGGCACAGCGAGAAGTCCGTGAGCCTTGCGAACTTGATACCCGCGAGTACGTTGCTGGTGCTCCTTACACCCCAGTTGAACATGTTGTGTTTAACCCTTCGTCTCGTGAcc                     <  1:321518/301‑1 (MQ=255)`  
                                                                                                                                                                                                                                                                                               `tAGAGTTAGCTTCTCGCCGCTCTGAGTTGCTCCGTAAATTGACCGAAACGTTCGGCTCGTGGTATCAGCCTAAAGGTGGCACTGAGATGTTCTGCCATCCGCGAACAGGTAAGCCACTACCTAAATACCCTCGCATTAAGACACCTAAAGTTGGTGGTATCTTTAAGAAGCCTAAGAACAAGGCACAGCGAGAAGGCCGTGAGCCTTGCGAACTTGATACCCGCGAGTACGTTGCTGGTGCTCCTTACACCCCAGTTGAACATGTTGTGTTTAACCCTTCGTCTCGTGACCACATTCagaa           <  1:62400/301‑1 (MQ=255)`  
                                                                                                                                                                                                                                                                                               `tAGAGTTAGCTTCTCGCCGCTCTGAGTTGCTCCGTAAATTGACCGAAACGTTCGGCTCGTGGTATCAGCCTAAAGGTGGCACTGAGATGTTCTGCCATCCGCGAACAGGTAAGCCACTACCTAAATACCCTCGCATTAAGACACCTAAAGTTGGTGGTATCTTTAAGAAGCCTAAGAACAAGGCACAGCGAGAAGGCCGTGAGCCTTGCGAACTTGATACCCGCGAGTACGTTGCTGGTGCTCCTTACACCCCAGTTGAACATGTTGTGTTTAACCCTTCGTCTCGTGACCACATTCagaa           <  1:128604/301‑1 (MQ=255)`  
                                                                                                                                                                                                                                                                                                `agagTTAGCTTCGCGCCGCTCTGAGTTGCTCCGTAAATTGACCGAAACGTTCGGCTCGTGGTATCAGCCTAAAGGTGGCACTGAGATGTTCTGCCATCCTCGAACAGGTAATCCACTACCTAAATACCCTCGCATTAAGACACCTAAAGTTGGTGGTATCTTTAAGAAGCCTAAGAACAAGGCACAGCGAGAAGGCCGTGAGCCTTGCGAACTTGATACCCGCGAGTACGTTGCTGGTGCTCCTTACACCCCAGTTGAACATGTTGTGTTTAACCCTTCGTCTCGTGACCACATTCagaag          <  1:328606/301‑1 (MQ=255)`  

                                                                                                                                                                                                                                                                                                          `|`                                                                                                                                                                                                                                                                                                            
`AGAGCAGGGTGAAGAATACGTTGACGGAATGGAGTGGTGGAACTTCAACGAAGAGATGATGGACTATAACGTTCAGGACGTTGTGGTAACTAAAGCTCTCCTTGAGAAGCTACTCTCTGACAAACATTACTTCCCTCCTGAGATTGACTTTACGGACGTAGGATACACTACGTTCTGGTCAGAATCCCTTGAGGCCGTTGACATTGAACATCGTGCTGCATGGCTGCTCGCTAAACAAGAGCGCAACGGGTTCCCGTTTGACACAAAAGCAATCGAAGAGTTGTACGTAGAGTTAGCTGCTCGCCGCTCTGAGTTGCTCCGTAAATTGACCGAAACGTTCGGCTCGTGGTATCAGCCTAAAGGTGGCACTGAGATGTTCTGCCATCCGCGAACAGGTAAGCCACTACCTAAATACCCTCGCATTAAGACACCTAAAGTTGGTGGTATCTTTAAGAAGCCTAAGAACAAGGCACAGCGAGAAGGCCGTGAGCCTTGCGAACTTGATACCCGCGAGTACGTTGCTGGTGCTCCTTACACCCCAGTTGAACATGTTGTGTTTAACCCTTCGTCTCGTGACCACATTCAGAAGAAACTCCA  >  NC_001604.1/14796‑15392`

  

Alignment Legend

Aligned base mismatch/match (shaded by quality score): `ATCG/ATCG` `< 3 ≤` `ATCG/ATCG` `< 11 ≤` `ATCG/ATCG` `< 24 ≤` `ATCG/ATCG` `< 35 ≤` `ATCG/ATCG` `< 38 ≤` `ATCG/ATCG`

Unaligned base: `atcg`    Masked matching base: `atcg`    Alignment gap: `‑`    Deleted base: `‑`
